# Dried plum consumption improves bone mineral density in osteopenic postmenopausal woman: A case report

**DOI:** 10.1016/j.bonr.2021.101094

**Published:** 2021-05-18

**Authors:** Nicole C.A. Strock, Kristen J. Koltun, Connie Weaver, Mary Jane De Souza

**Affiliations:** a Penn State University, University Park, PA, USA; b Weaver and Associates Consulting, LLC, USA

**Keywords:** Osteopenia, Osteoporosis, Dried plum, Bone mineral density, Menopause

## Abstract

The use of non-pharmacological alternatives to pharmacological interventions, e.g., nutritional therapy, to improve or maintain bone mineral density (BMD) in postmenopausal women has gained traction over the past decade, but limited data exist regarding its efficacy. The purpose of this case report was to compare changes in BMD of an osteopenic postmenopausal woman over the course of 28 months, including an abrupt change in diet. For the first 12 months, a participant assigned to the control arm of a randomized controlled trial (RCT) only took calcium and vitamin D_3_ supplements, but in the following 16 months after completing the RCT, she introduced and maintained daily consumption of 50 g of dried plums in addition to calcium and vitamin D_3_ supplements. This case report provides a unique opportunity to follow the trajectory of distinct changes in bone in response to one dietary modification.

## 1 Introduction

Osteoporosis, a condition characterized by low bone mass and compromised bone quality contributing to decreased bone strength and increased risk of fracture (NIH Consensus Development Panel on Osteoporosis Prevention, D. and Therapy, 2001), poses a threat not only to the aging individual who faces increased bone fragility and fracture risk, but also contributes substantially to global healthcare costs. In the U.S., it is estimated that osteoporosis will impact 13.6 million women 50 years and older with the prevalence of low bone mass expected to reach 57.8 million by 2030 (Wright et al., 2014). Additionally, more than 2 million fragility fractures occur annually in the U.S., incurring health costs that exceed 15 billion dollars per year (Burge et al., 2007). The projected 50% increase in yearly cost and incidence of fragility fractures by 2025 (Burge et al., 2007) underscores the urgency for continued investigation and improvement of preventative strategies.

Numerous pharmacological therapies can effectively treat bone loss and osteoporosis, including hormone therapy, bisphosphonates, selective estrogen receptor modulators, RANK ligand inhibitors, and sclerostin inhibitors (Barrionuevo et al., 2019). However, in order to avoid the undesirable side effects of certain pharmacological therapies, women are seeking alternative non-pharmacological therapies (Kurzer, 2003). The use of non-pharmacological interventions (e.g., nutritional therapy) to improve or maintain bone mineral density (BMD) in postmenopausal women has gained traction over the past decade (Sansai et al., 2020; Thaung Zaw et al., 2018). While supplementation of calcium and vitamin D_3_ is considered the minimal standard of care for postmenopausal women with low BMD due to its modest benefits on bone health (Weaver et al., 2016a, Weaver et al., 2016b), animal research investigating plant-derived compounds, such as the phenolic compounds (chlorogenic acids, phenolic acids, and flavonoids (Treutter et al., 2012; Mubarak et al., 2012)) found in fruits (e.g. dried plums and blueberries) indicate promising potential for nutraceuticals that may positively impart benefits to bone (Arjmandi, 2001; Deyhim et al., 2005; Franklin et al., 2006; Muhlbauer et al., 2003; Pawlowski et al., 2014; Chen et al., 2019; Zhao et al., 2020). With respect to dried plums, it is proposed that the phenolic compounds may mitigate the negative effects of oxidative stress (Mubarak et al., 2012) and target inflammatory pathways (Franklin et al., 2006) that are upregulated in a hypoestrogenic environment and mediate negative effects on bone. Specifically, rodent models have demonstrated decreases in bone resorption, increases in bone formation, and improvements in bone microarchitecture and bone trophic hormones following supplementation with dried plums (Arjmandi, 2001; Deyhim et al., 2005; Franklin et al., 2006; Muhlbauer et al., 2003).

However, limited investigations regarding the efficacy and dosage of whole dried plum supplementation on BMD and bone metabolism outcomes in humans currently exist. To date, the results from two randomized controlled trials (RCT) investigating the effects of whole dried plum consumption on bone health outcomes in postmenopausal women (Arjmandi et al., 2002; Hooshmand et al., 2011, Hooshmand et al., 2016, Hooshmand et al., 2014), indicate promising effects on bone biomarkers (Arjmandi et al., 2002) and BMD (Hooshmand et al., 2011, Hooshmand et al., 2016). Of the results from shorter duration investigations, 3 months of 100 g dried plum increased serum makers of formation (Arjmandi et al., 2002), while 6 months of either 50 g or 100 g dried plum decreased markers of resorption (Hooshmand et al., 2016). In the 12-month RCT, 100 g dried plum improved BMD at the ulna and spine, while decreasing select serum markers of bone turnover and resorption compared to 75 g dried apples (Hooshmand et al., 2011, Hooshmand et al., 2014). Notably, while both 100 g dried plum and 75 g dried apple groups received daily calcium and vitamin D (Hooshmand et al., 2011, Hooshmand et al., 2014), it is currently unknown whether various dried plum dosages yield comparable or improved effects on BMD and bone metabolism during a 12-month investigation.

Currently, our lab is conducting a 12-month RCT assessing the dose-dependent effects (i.e., 0 vs 50 g vs 100 g of dried plum) of daily dried plum consumption on bone health outcomes in postmenopausal women with low BMD. However, RCT results can be slow to develop due to the extended timing of the intervention, data analysis, and publication. Therefore, this case report provides intriguing preliminary findings and provides a unique circumstance in which one participant completed both 1) the control arm of the RCT for 12 months and 2) subsequently consumed 50 g dried plum for 16 months allowing us to investigate the effect of dried plum on bone in an individual who acted as her own control. The purpose of this case report was to compare changes in BMD of an osteopenic woman over the course of 28 months; 12 months involved consuming only calcium and vitamin D_3_ supplements followed by 16 months of daily consumption of 50 g of dried plums in addition to calcium and vitamin D_3_ supplements.

## 2 Case presentation

**Fig. 1:**
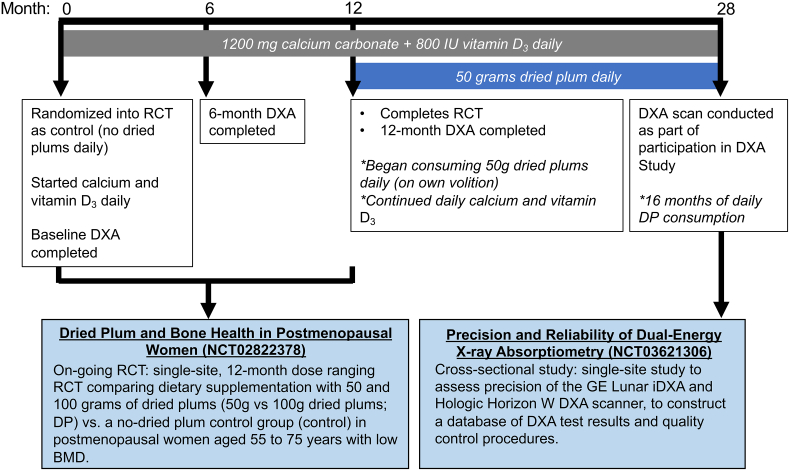
Timeline of bone mineral density (BMD) measures in osteopenic postmenopausal woman, who participated in two separate studies performed in the same laboratory. DXA, dual-energy X-ray absorptiometry; RCT, randomized controlled trial; DP, dried plum.

A 55-year old woman participated in a 12-month RCT investigating the effects of dried plum consumption in postmenopausal women (Dried Plum and Bone Health in Postmenopausal Women, NCT02822378) conducted by the Women's Health and Exercise Laboratory at Pennsylvania State University. As part of the RCT, the subject was randomly assigned to the control group (no dried plums) and prescribed 1200 mg calcium carbonate and 800 IU vitamin D_3_ daily for 12 months, during which dual-energy x-ray absorptiometry (DXA) scans were repeated at 6 and 12 months for comparison to baseline. Following study completion, and of her own volition, the participant began consuming 50 g (approximately 6) dried plums daily, in addition to maintaining the previously prescribed calcium/vitamin D_3_ supplements. Sixteen months after completing her participation in the RCT (28 months after baseline measurement), and during which she voluntarily maintained the aforementioned daily dried plum 50 g dosing and calcium/vitamin D_3_ intake, the participant enrolled in a different study being conducted in the same laboratory (DXA Study of Precision and Reliability NCT03621306), in which a DXA scan was performed (Fig. 1). All DXA scans performed during both studies were obtained on the same Hologic QDR4500 system, allowing for longitudinal BMD comparison. Intra-operator precision is as follows: lumbar spine: 0.84% (LSC = 2.33%), total hip: 0.79% (LSC = 2.18%). Scans were analyzed by the same International Society for Clinical Densitometry certified bone densitometry technologist, for consistency.

Upon entry to the RCT, the woman was 5 years post-menopause, with no history of menstrual disturbances, premature ovarian failure, estrogen or hormone therapy use, osteoporosis medication use, fragility fracture, or familial history of osteoporosis. The woman reported no alcohol consumption and had a history of smoking for 1-year duration from age 15 to 16. A baseline DXA scan for the RCT indicated osteopenia (Total Body BMD: 1.021 g/cm^2^, T-score: −1.1; L1-L4 spine BMD: 0.871 g/cm^2^, T-score: −1.6; total hip BMD: 0.817 g/cm^2^, T-score: −1.0). During months 0–12 when calcium and vitamin D_3_ were consumed, DXA scans were repeated at 6 and 12 months.

**Fig. 2:**
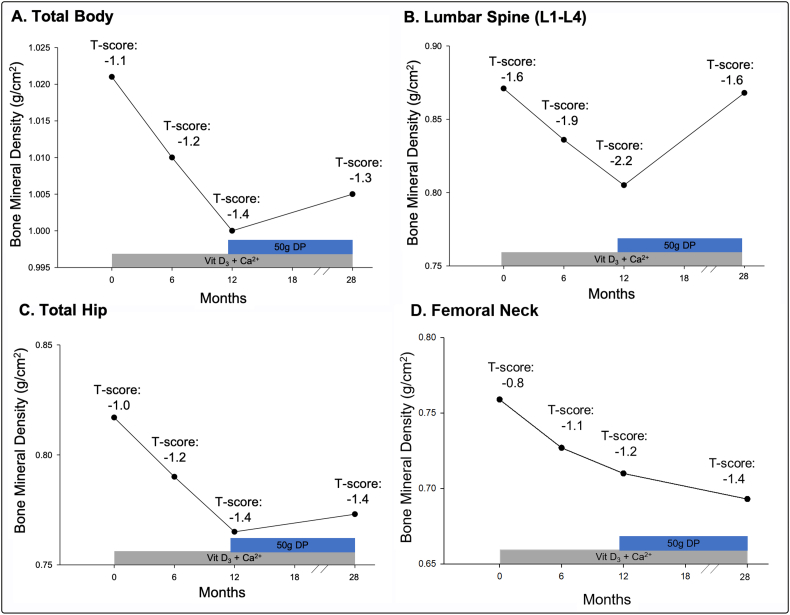
Changes in bone mineral density over 28-month period at A) total body, B) lumbar spine (L1-L4), C) total hip, and D) femoral neck. From months 0–12, participant supplemented with 1200 mg calcium carbonate (Ca^2+^) and 800 IU vitamin D_3_ daily, as part of control group for on-going 12-month randomized controlled trial (RCT). Upon completion of RCT, participant introduced 50 g of dried plums (DP) daily, in addition to maintaining calcium and vitamin D_3_ supplementation. T-score at each time point superimposed. T-score < −1.0: osteopenia.

Following 12 months of calcium and vitamin D_3_ supplementation, total body BMD was reduced by 2.1% (BMD: 1.000 g/cm^2^, T-score: −1.4), lumbar spine (L1-L4) BMD was reduced by 7.6% (BMD: 0.805 g/cm^2^, T-score: −2.2), total hip BMD was reduced 6.4% (BMD: 0.765 g/cm^2^; T-score: −1.4), and femoral neck was reduced 6.5% (BMD: 0.710 g/cm^2^; T-score: −1.2). BMD and body composition over the study duration are described in Fig. 2 and Table 1, respectively. However, from months 12 to 28, which included daily consumption of 50 g dried plum daily in addition to calcium/vitamin D_3_ supplementation, total body BMD increased by 0.5% (total body BMD: 1.005 g/cm^2^, T-score: −1.3), lumbar spine BMD increased by 7.8% (L1-L4 BMD: 0.868 g/cm^2^, T-score: −1.6), total hip BMD increased by 1.05% (total hip BMD: 0.773 g/cm^2^; T-score: −1.4), and femoral neck decreased by 2.4% (femoral neck BMD: 0.693 g/cm^2^; T-score: −1.4). At month 28, lumbar spine BMD was within 0.5% of baseline values, essentially recovering the BMD lost as a control participant in the RCT, while total body, total hip, and femoral neck BMD did not improve compared to baseline values. The trajectory of BMD over the 28-month time frame is visualized in Fig. 2.

**Table 1:** Anthropometric characteristics measured over the course of 28 months. Baseline, 6 month, and 12 month measures were conducted while participating in Dried Plum (DP) RCT, when randomized into the control group (consuming 0 dried plums daily). Final measure was conducted during participation in Precision and Reliability DXA study, 28 months after baseline measure and after voluntarily consuming 50 g DP daily for 16 months. Exercise minutes were self-reported. BMI, body mass index; IGF-1, insulin-like growth factor 1.

Variable	Dried Plum RCT participation (control group)			16 months of 50 g DP
	Baseline	6 month	12 month	28 months after baseline
Age (yr)	55.5	56.0	56.7	58.0
Age of menarche (yr)	12	–	–	–
Age of menopause (yr)	50	–	–	–
Height (cm)	166.4	–	–	166.1
Body mass (kg)	60.8	62.3	62.0	62.7
BMI (kg/m^2^)	22.6	22.8	22.6	23.0
Fat mass (kg)	14.9	16.6	15.5	15.2
Body fat (%)	24.5	26.7	25.0	24.2
Lean mass (kg)	43.9	43.6	44.6	45.5
Fat free mass (kg)	45.9	45.7	46.5	47.5
IGF-1 (ng/mL)	127.8	–	156.3	–
Vitamin D (ng/mL)	29.0	30.7	36.3	–
Exercise (minutes/week)	317	260	180	–

## 3 Discussion

This case study represents a unique investigation, in which BMD of an osteopenic postmenopausal woman was monitored over the course of 28 months. Specifically, daily consumption of calcium and vitamin D_3_ supplements during months 1–12 did not prevent consistent decrements in BMD over a 12-month period, with the most drastic decline in the lumbar spine. The bone loss at the lumbar spine of 7.6% as a control participant in the RCT far exceeded bone loss previously reported in a large cohort of postmenopausal women (2.0%) (Finkelstein et al., 2008), despite receiving calcium and vitamin D_3_ supplementation, considered the minimal standard of care for postmenopausal women with low BMD (Weaver et al., 2016a, Weaver et al., 2016b). Interestingly, long term consumption (i.e., 16 months) of 50 g dried plum in conjunction with calcium/vitamin D_3_ essentially reversed the BMD decrement observed during months 1–12 in the lumbar spine, the most common site for fragility fracture (Ensrud, 2013), while preventing further decline in total body and total hip BMD. These data are consistent with previous RCT findings demonstrating the bone health benefits of dried plum consumption in osteopenic postmenopausal women (Hooshmand et al., 2011, Hooshmand et al., 2016). In such investigations, dried plum consumption prevented BMD loss at the total body (Hooshmand et al., 2016) over 6 months, and improved ulna and spine BMD over 12 months (Hooshmand et al., 2011). Notably, the improvement to spine BMD in the 16 months (7.8%) following participation as a control in the RCT exceed improvements previously reported in women consuming double the daily dosage (100 g) of dried plums (Hooshmand et al., 2011). It should be noted that during the 16 months of volitional dried plum consumption, no physical activity or dietary records were maintained. Together, this investigation suggests that a non-pharmacologic dietary intervention of 50 g dried plum daily for at least 12 months may positively impact BMD, better than calcium and vitamin D_3_ supplementation alone, in postmenopausal women with osteopenia.

## Funding source

This work was supported by the 10.13039/100011024California Dried Plum Board.

## Informed consent

Informed Consent was obtained for both studies in which the participant took part.

## Authorship

Nicole Strock: conceptualization, data curation, writing- original draft.

Kristen Koltun: data curation, writing- review & editing.

Connie Weaver: supervision, writing- review & editing.

Mary Jane De Souza: supervision, writing- review & editing.

## Declaration of competing interest

Dr. Weaver is currently on the California Dried Plum Board, but was not on the board during the course of this investigation. All other authors declare that they have no conflicts of interest.
